# Supplementary material for: Influenza A deletion-containing viral genome production is altered by metabolites, metabolic signaling molecules, and cyanobacterial extracts
Source: Appl Environ Microbiol. 2026 Aug 20;92(9):e01232-26. doi: 10.1128/aem.01232-26 (PMC13599498; doi:10.1128/aem.01232-26)
Supplement: Supplementary file 1 [file aem.01232-26-s0001.pdf]

SUPPLEMENTARY MATERIALS

Influenza A defective viral genome production is altered by metabolites, metabolic signaling molecules, and cyanobacteria extracts

Ilechukwu Agu[1], Ivy R. José[1], Samuel L. Díaz-Muñoz[1,2*]

Extended Results

Drugs affect DVG proportion and TVG production at the segment level for CA09 and TX12

Continued from Results in the main text, we report the segment-specific effects of drug treatments on DelVG proportion and total genomes that were significant relative to their vehicle control (DMSO, dH_2O). As stated, we analyzed differences among treatments using ANOVA, adding bioreplicate, strain, and segment as covariates.

Strain	Vehicle	Measure	Segment	Treatment	Estimate	Std. Error	t value	Pr(>|t|)	
**CA09**	H2O	PropDVG	PB1	TxAdo	0.575611	0.204348	2.817	1.24E-02	*
**CA09**	H2O	PropDVG	PB2	TxAdo	0.350356	0.10737	3.263	0.00488	**
**CA09**	H2O	PropDVG	PA	TxAdo	0.514519	0.140622	3.659	0.00212	**
**TX12**	H2O	PropDVG	PB1	TxAdo	0.80981	0.20321	3.985	1.06E-03	*
**TX12**	H2O	PropDVG	PB2	TxAdo	0.603432	0.122571	4.923	0.000153	**
**TX12**	H2O	PropDVG	PA	TxAdo	0.78621	0.178854	4.396	0.000451	***
**TX12**	H2O	PropDVG	NP	TxAdo	0.009966	0.0036557	2.726	0.014943	*
**CA09**	H2O	PropDVG	HA	TxAdo	0.337257	0.1237154	2.726	0.015	*
**TX12**	H2O	PropDVG	PB1	TxInsu	0.52964	0.20321	2.606	0.01909	*
**TX12**	H2O	PropDVG	PA	TxInsu	0.387404	0.178854	2.166	0.045756	*
**TX12**	H2O	PropDVG	PB2	TxInsu	0.365633	0.122571	2.983	0.008785	**
**TX12**	H2O	PropDVG	NP	TxInsu	0.007798	0.0036557	2.133	0.048746	*
**TX12**	DMSO	PropDVG	NA	TxFavp	-0.01216	0.0047605	-2.556	0.0286	*
**TX12**	H2O	PropDVG	NA	TxLepto	-0.01946	0.004713	-4.13	0.001021	**
**TX12**	DMSO	PropDVG	NA	TxMK2206	0.014262	0.0047605	2.996	0.0134	*
**TX12**	H2O	PropDVG	NA	TxTolyp	-0.01150	0.004713	-2.441	0.028536	*
**BOTH**	DMSO	TVG	PB2	Tx4-OI	2287.3	905.6	2.526	0.0177	*
**BOTH**	DMSO	TVG	PB1	Tx4-OI	1177.67	462.2	2.548	0.0168	*
**BOTH**	DMSO	TVG	PA	Tx4-OI	3886.3	1285.5	3.023	0.00543	**
**BOTH**	DMSO	TVG	HA	Tx4-OI	8590	2447.24	3.51	0.00159	**
**BOTH**	DMSO	TVG	NP	Tx4-OI	9482.83	2792.07	3.396	0.00213	**
**BOTH**	DMSO	TVG	NA	Tx4-OI	11798.3	3463.2	3.407	0.00207	**
**BOTH**	DMSO	TVG	M	Tx4-OI	30476	8857	3.441	0.0019	**
**CA09**	DMSO	TVG	NS	Tx4-OI	33209	12276	2.705	0.0221	*
**BOTH**	DMSO	TVG	HA	TxUK5099	5051.67	2447.24	2.064	0.04873	*
**BOTH**	DMSO	TVG	NP	TxUK5099	6189.17	2792.07	2.217	0.03526	*
**BOTH**	DMSO	TVG	NA	TxUK5099	7577.3	3463.2	2.188	0.0375	*
**BOTH**	DMSO	TVG	M	TxUK5099	20742	8857	2.342	0.0268	*
**BOTH**	DMSO	TVG	PA	TxFavp	2767.7	1285.5	2.153	0.04041	*
**BOTH**	DMSO	TVG	NP	TxFavp	5809.5	2792.07	2.081	0.04708	*
**BOTH**	DMSO	TVG	M	TxFavp	19298	8857	2.179	0.0382	*

**TX12**	H2O	TVG	HA	TxLepto	-4135.67	1052.26	-3.93	0.001336	**
**TX12**	H2O	TVG	NA	TxLepto	-5725.33	1824.47	-3.138	0.00726	**
**TX12**	H2O	TVG	PB1	TxTolyp	1410	532.93	2.646	0.0176	*
**TX12**	H2O	TVG	HA	TxTolyp	-3178	1052.26	-3.02	0.008611	*
**TX12**	H2O	TVG	NA	TxTolyp	-4163.67	1824.47	-2.282	0.03864	*

Supplemental Table 1. **Drugs significantly alter total viral genomes and proportion of deletion containing viral genomes at the segment level**. Segment-specific statistically significant predictors of the proportion of **Del**etion-containing **V**iral **G**enomes (DelVGs) and **T**otal **V**iral **G**enomes (TVG) and their parameter estimates from ANOVA. The asterisks in the final column indicate p-values ranges: * = $p < 0.05$, ** = $p < 0.01$, *** = $p < 0.001$.

Drugs affect DVG proportion and TVG Production at the genome level for CA09 and TX12

Below are visualizations of DVG relative abundance (**Supplementary Figure 1**) and total viral genomes (**Supplementary Figure 2**) for CA09 and TX12 *at the genome level*, averaged across three bioreplicates.

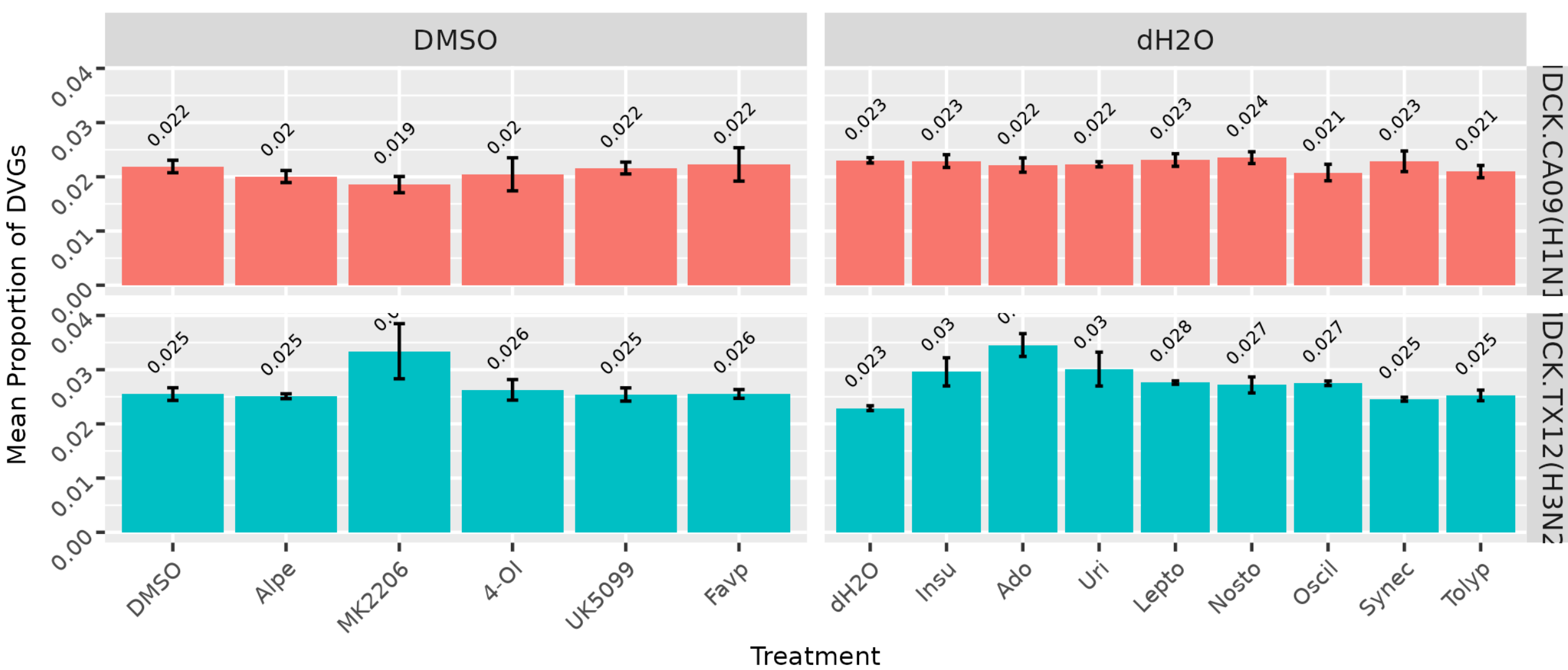

Supplemental Figure 1. Mean proportion of total viral genomes (CA09/TX12) that are DVGs after 18 h.p.i. under different treatment conditions; no trypsin. Vehicle treatment groups received either DMSO or dH_2O treatment. n = 3 bioreplicates, sem.

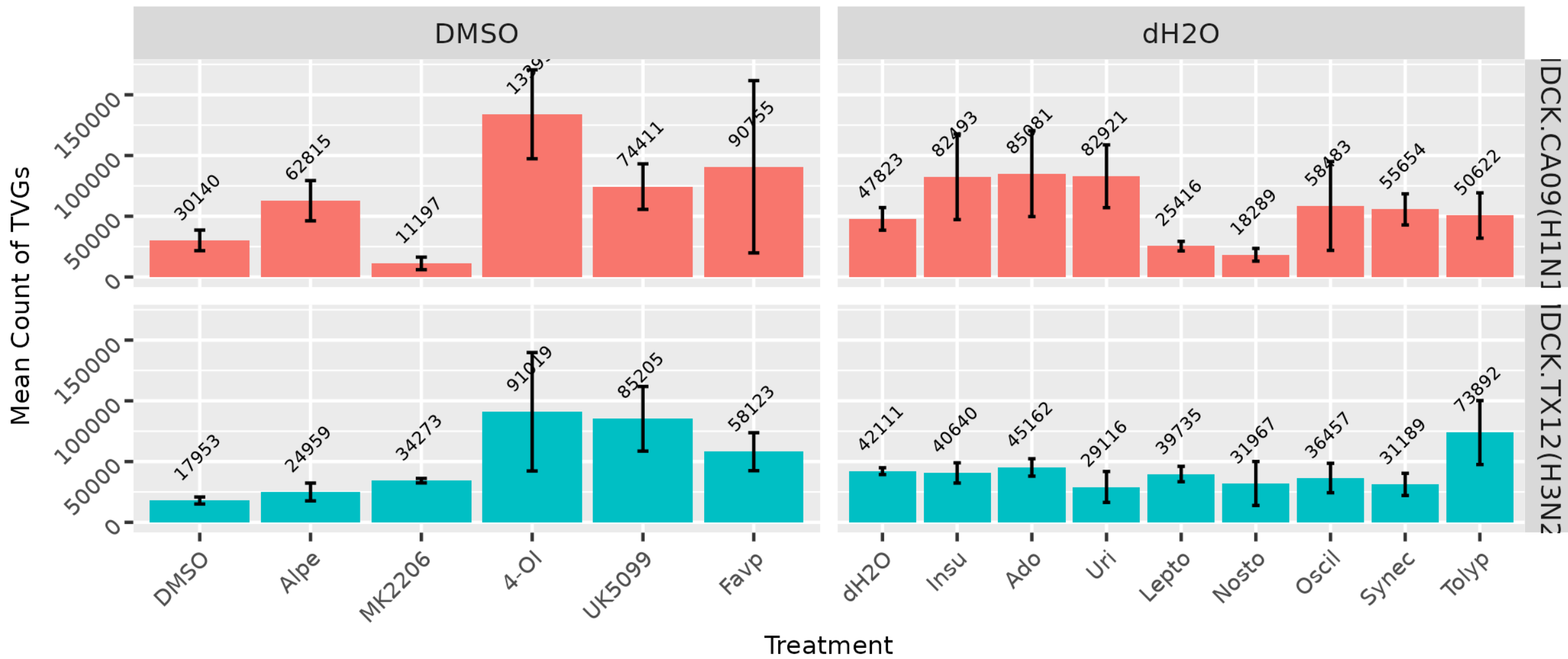

Supplemental Figure 2. Mean count of total viral genomes (CA09/TX12) recovered at 18 h.p.i. under different treatment conditions; no trypsin. Vehicle treatment groups received either DMSO or dH_2O treatment. n = 3 bioreplicates, sem.

3A

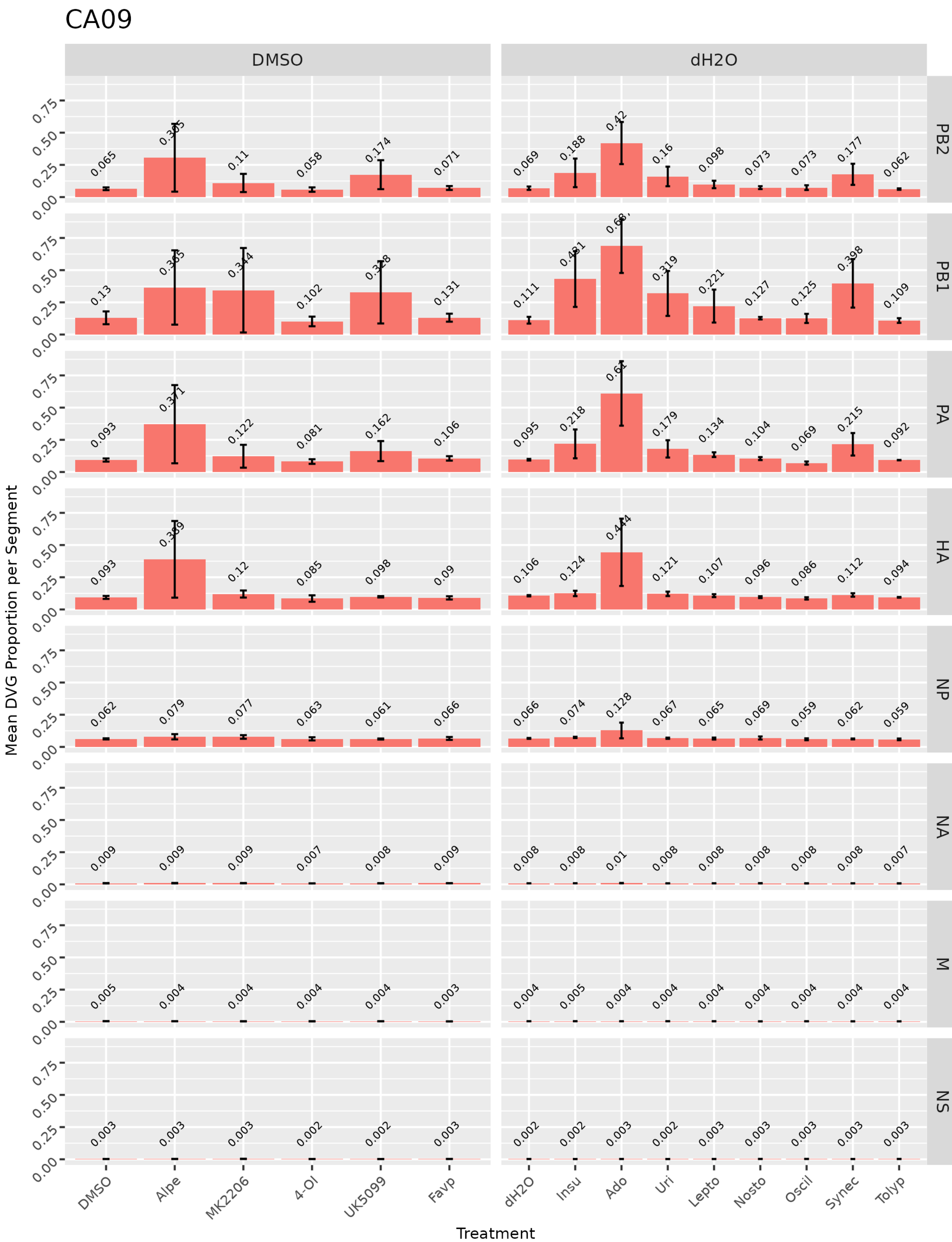

CA09
DMSO
dH2O
PB2
PB1
PA
HA
NP
NA
M
NS
Mean DVG Proportion per Segment
0.00
0.25
0.50
0.75
DMSO
Alpe
MK2206
4-OI
UK5099
Favp
dH2O
Insu
Ado
Uri
Lepto
Nosto
Oscil
Synec
Tolyp
Treatment

3B

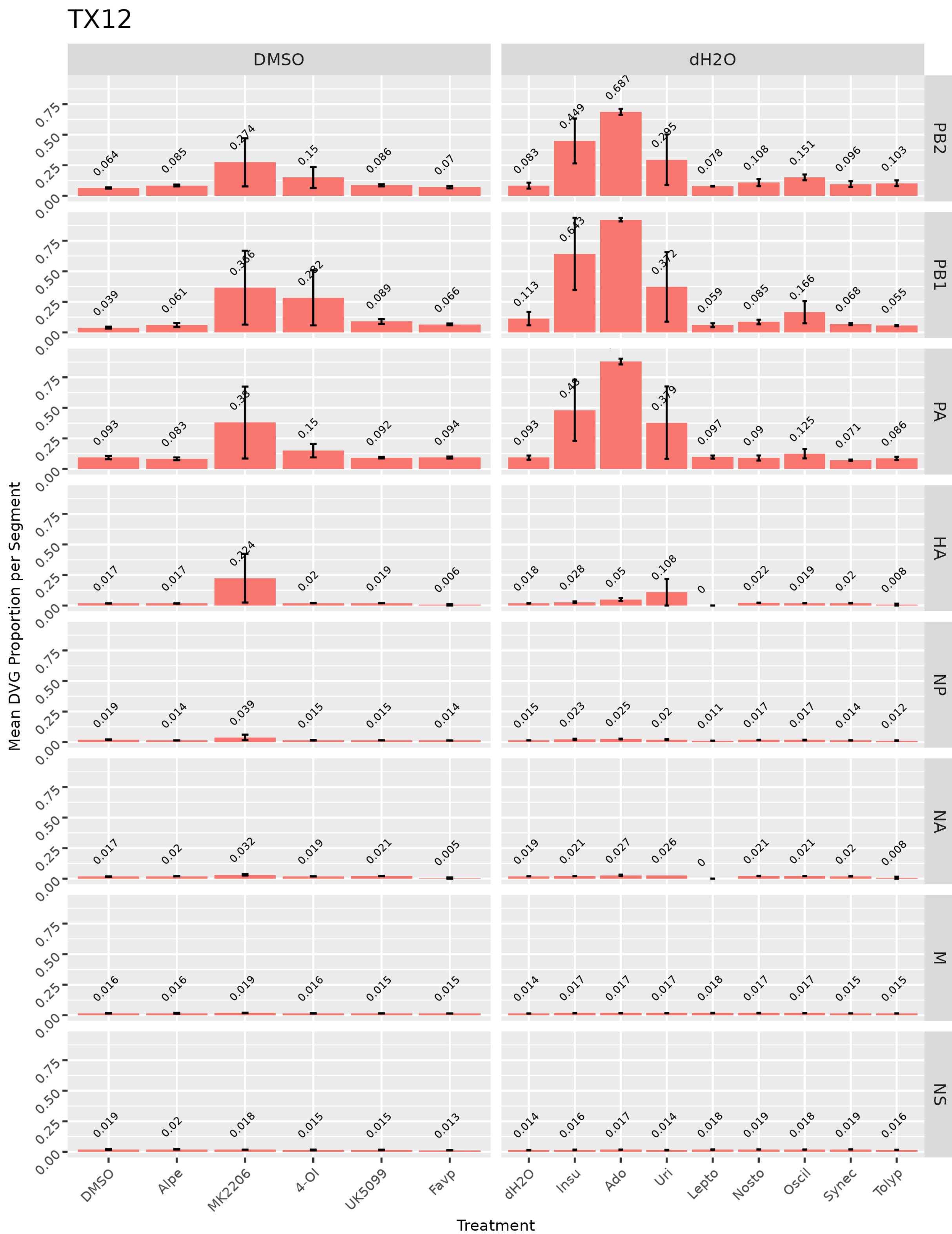

Supplementary Figure 3. Mean proportion of total viral genomes per segment that are DVGs at 18 h.p.i. under different treatment conditions; no trypsin. (**A**) CA09. (**B**) TX12. Vehicle treatment groups received either DMSO or dH_2O treatment. n = 3 bioreplicates, sem.

3.4A

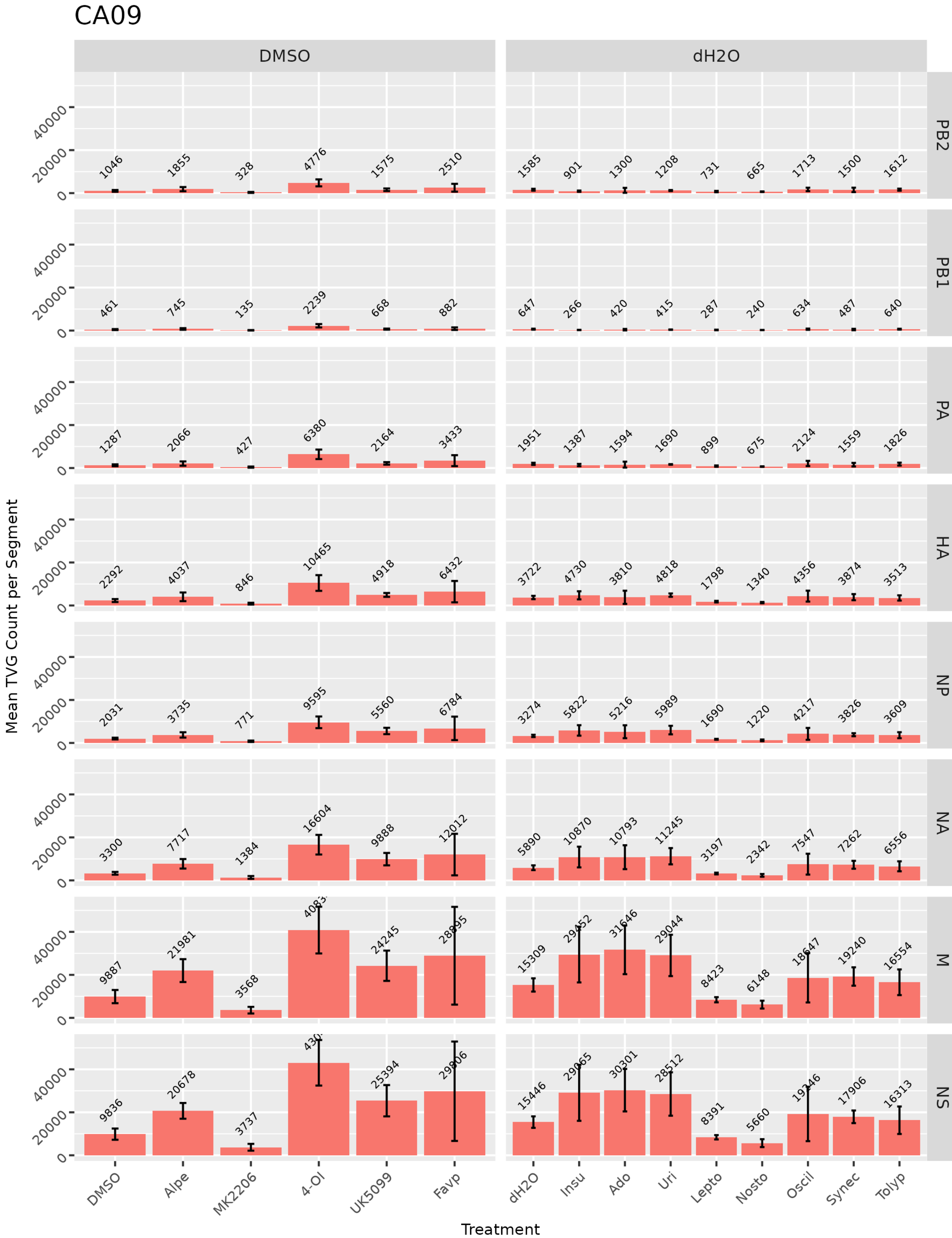

CA09
DMSO
dH2O
PB2
PB1
PA
HA
NP
NA
M
NS
Mean TVG Count per Segment
Treatment
DMSO
Alpe
MK2206
4-OI
UK5099
Favp
dH2O
Insu
Ado
Uri
Lepto
Nosto
Oscil
Synec
Tolyp

3.4B

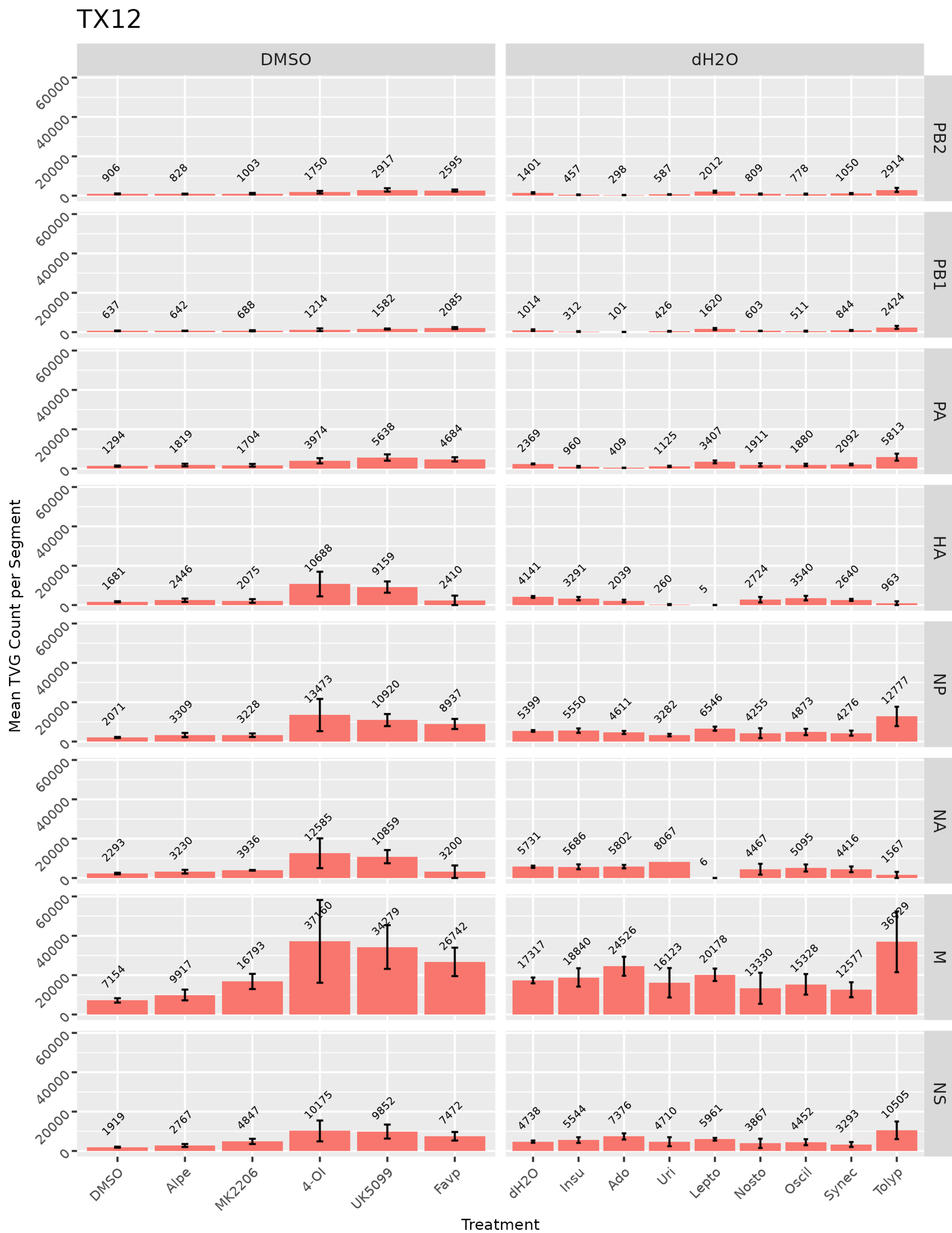

Supplementary Figure 4. Mean count of total viral genomes per segment produced after 18 h.p.i. under different treatment conditions; no trypsin. (**A**) CA09. (**B**) TX12. Vehicle treatment groups received either DMSO or dH_2O treatment. n = 3 bioreplicates, sem.

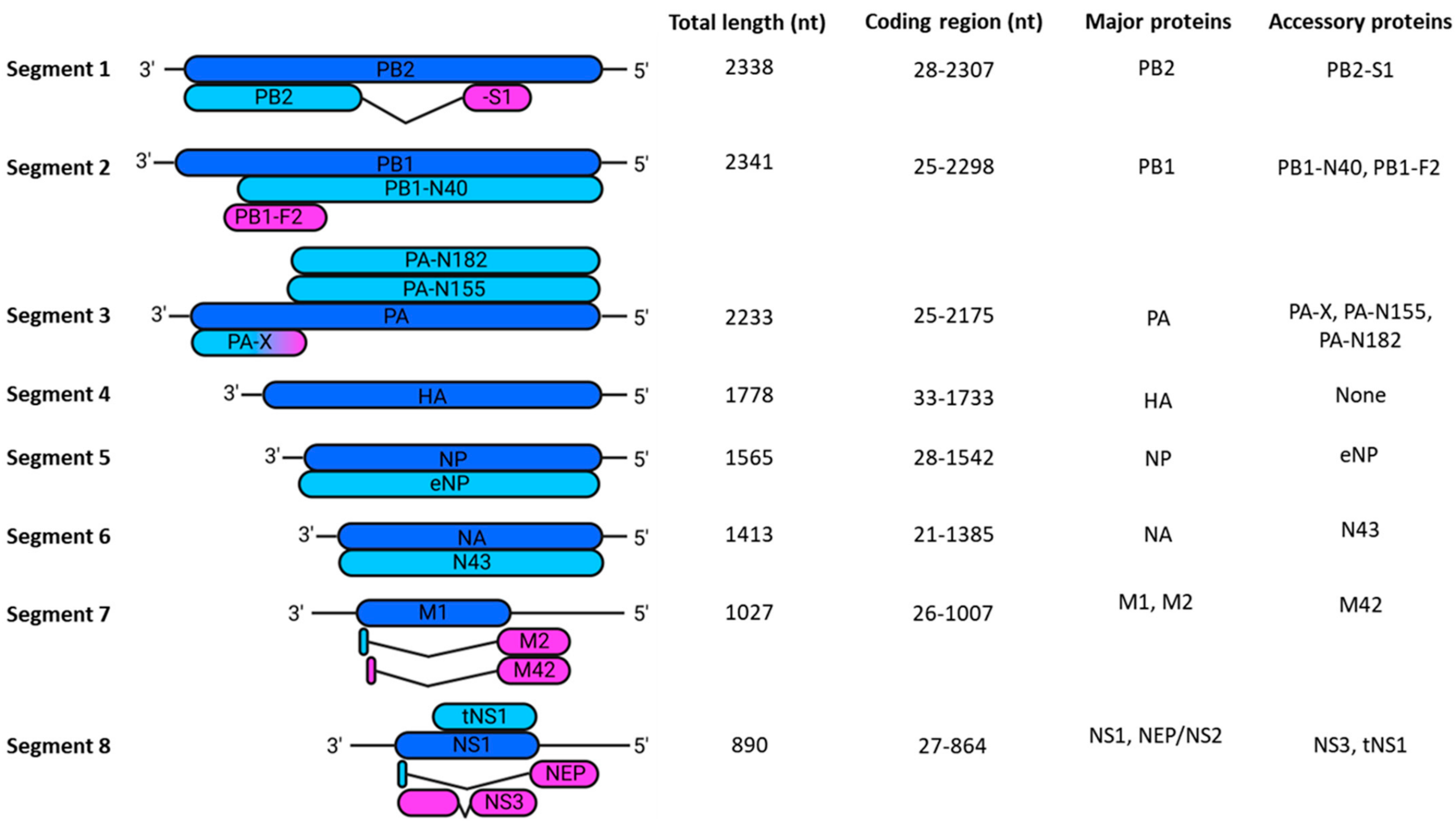

Supplementary Figure 5. Schematic representation of influenza A viral genome segments. The eight influenza A viral genome segments are depicted and accompanied by length (in strain A/Puerto Rico/8/1934 (H1N1)), coding regions, major and accessory proteins. In each segment: black lines = non-coding regions; dark blue = frame 1 major proteins; light blue = frame 1 accessory proteins; magenta = frame 2 accessory proteins, diagonal lines = mRNA splicing. Figure is Figure 2 from Carter T, Iqbal M. (2024) The Influenza A Virus Replication Cycle: A Comprehensive Review. Viruses. 2024 Feb 19;16(2):316. doi: 10.3390/v16020316, reproduced here under CC-BY license.

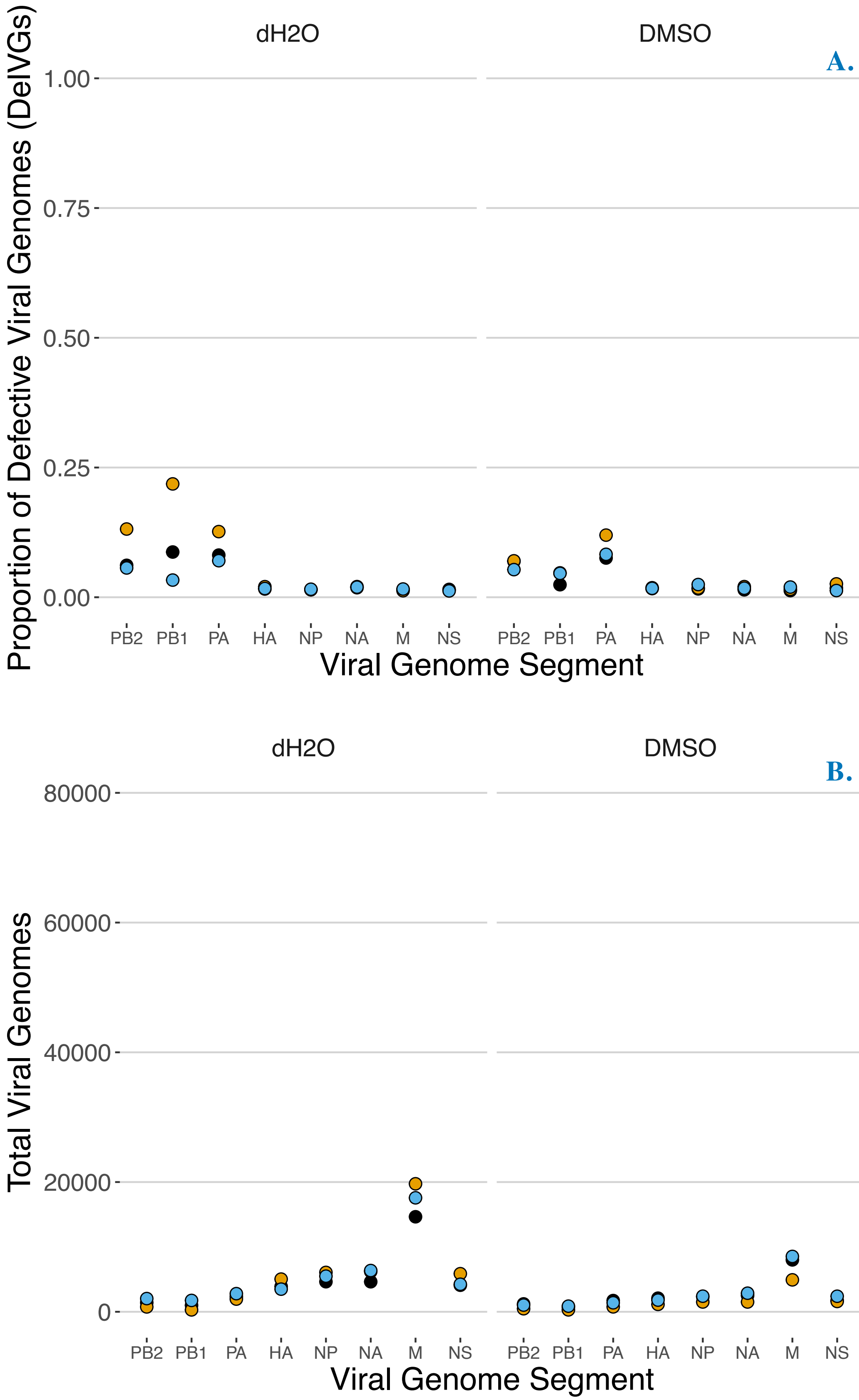

Supplementary Figure 6. Control infections were highly repeatable. A. Production of influenza defective viral genomes (here measured as proportion of Deletion-containing Viral Genomes (DelVGs) in strain TX12 (H3N2) after 18 hr infection (no trypsin) mock-treated with water vehicle (left panel) and DMSO vehicle (right panel). B. Production of influenza Total Viral Genomes (TVG) in strain TX12 (H3N2) after 18 hr infection (no trypsin) mock-treated with water vehicle (left panel) and DMSO vehicle (right panel). Point colors represent data from three independent infections conducted on different days (i.e. bioreplicates).

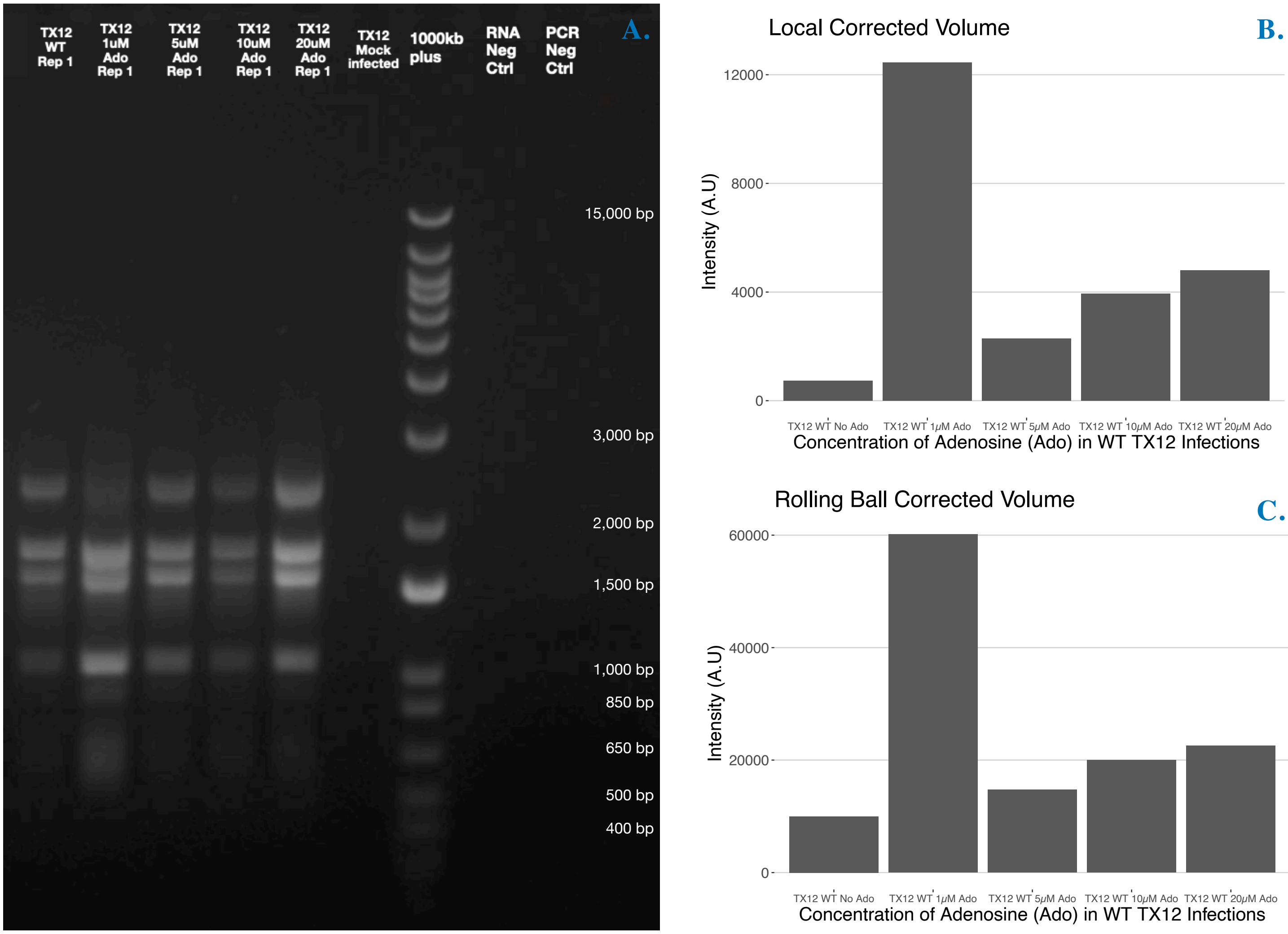

Supplementary Figure 7. Adenosine increases DelVGs in TX12 WT stocks with pre-existing DelVGs. A. Gel showing whole genome, multi-segment amplification of infection supernatants of wildtype egg-passaged TX12 stock under different adenosine concentrations. Infections were TX12 (H3N2) after 18 hr (no trypsin), as in the rest of the text. The last band in the gel shows the DelVGs. **B & C.** Intensity (A.U.) of the DelVG band as a function of adenosine concentration, as calculated by GelGenie (Aquilina et al. 2025). Top plot shows quantification using the local corrected volume (**B.**), bottom plot shows quantification using the rolling ball corrected volume (**C.**).
